# Truthfulness in transplantation: non-heart-beating organ donation

**DOI:** 10.1186/1747-5341-2-17

**Published:** 2007-08-24

**Authors:** Michael Potts

**Affiliations:** 1 Methodist University, Fayetteville, NC 28311-1498 USA

## Abstract

The current practice of organ transplantation has been criticized on several fronts. The philosophical and scientific foundations for brain death criteria have been crumbling. In addition, donation after cardiac death, or non-heartbeating-organ donation (NHBD) has been attacked on grounds that it mistreats the dying patient and uses that patient only as a means to an end for someone else's benefit.

Verheijde, Rady, and McGregor attack the deception involved in NHBD, arguing that the donors are not dead and that potential donors and their families should be told that is the case. Thus, they propose abandoning the dead donor rule and allowing NHBD with strict rules concerning adequate informed consent. Such honesty about NHBD should be welcomed.

However, NHBD violates a fundamental end of medicine, nonmaleficience, "do no harm." Physicians should not be harming or killing patients, even if it is for the benefit of others. Thus, although Verheijde and his colleages should be congratulated for calling for truthfulness about NHBD, they do not go far enough and call for an elimination of such an unethical procedure from the practice of medicine.

## Commentary

In recent years, there has been a refreshing display of honesty regarding the current practice of organ transplantation. The basis for considering "brain dead" individuals to be truly dead has undergone a scathing scientific and philosophical critique [1-3]. Non-heartbeating organ donation (NHBD; a more accurate term than "donation after cardiac death" since two to five minutes of cardiac arrest is not sufficient for the heart to be "dead") is another dubious attempt by the transplant community to increase the organ supply. In NHBD protocols, patients with such conditions as "irreversible brain injury, end-stage musculoskeletal disease and high spinal cord injury" [4] are removed from life support. After cardiac arrest ensues, a period of time is allowed to pass, usually five minutes. After that time period, it is believed that autoresuscitation is impossible, and these patients are declared dead by cardiac criteria even if they do not meet brain death criteria [4]. After that time period, organ procurement surgery commences.

Shortly after the introduction of NHBD with the "Pittsburgh Protocol," Renee Fox argued that the practice was "an ignoble form of cannabilism" [5]. She objects to a procedure of controlled death, with the dying person isolated from friends and family members. She also opposes pharmacological support for dying organs that is not designed to benefit the donor, but only uses the donor in a strictly utilitarian way for the benefit of another person.

The concerns of Verheijde, Rady, and McGregor [6] relate to honesty in informing the public of what is really going on in NHBD. They correctly note that declaring such donors dead is a fiction that ignores the possibility of autoresuscitation as well as the fact that the brains of these patients are not truly dead. They call for a change in current organ donation policy that would eliminate the dead donor rule, allow NHBD, but only in the context of potential donors and their families receiving sufficient information to make a truly informed consent to the procedure. Since their proposed policy admits that such donors are not dead, it does not involve the deception of declaring them dead after a limited period of cardiac arrest. The public will know that it is the process of organ donation that results in their loved one's death and that heparin, phentolamine (used to prevent clotting and maintain perfusion [7]) and other drugs designed to prevent organ damage are not for the benefit of the donor and could theoretically hasten death [4,7]. If a person or family member desires to give consent for organ donation in these circumstances, at least that person will realize what he or she is authorizing.

If NHBD continues, then such truthfulness is better than deception. However, the authors' conclusions would allow NHBD to continue. Even with truthfulness and real informed consent, NHBD is unethical and should not be a part of medical practice. The principle of nonmaleficence ("do no harm") is essential to the good practice of medicine. Physicians have a great deal of knowledge about life and death, as well as the power to use such knowledge for good or ill. Some temporary harm to a patient (as in the side-effects of chemotherapy for cancer) are acceptable only because there is overall benefit to the patient. Procedures that can only cause harm to a patient without providing any benefit are unethical and the person performing them is no longer practicing medicine.

NHBD involves giving the donor drugs that preserve the donor's organs, but may hasten the donor's death. They are not therapeutic for the patient; they are given for utilitarian reasons for someone else's benefit. Since the patient is not truly dead until his or her organs are removed, it is the process of organ donation itself that causes the donor's death. Harming or killing a patient, even for the benefit of others, is an abuse of power and a violation of the trust that patients must have in medicine that doctors will help them, not harm them. It is unfortunate that the organ transplant community has allowed utilitarian considerations to override one of the fundamental ends of medicine, to help a patient in need and not harm that patient. While discontinuing NHBD would restrict the range of acceptable organ donation, maintaining an immoral practice is not worth the price. NHBD is an abuse of medicine, and should, therefore, be banned from the practice of medicine.
